# Is a high preoperative HbA1c level a risk factor for postoperative complications in patients with non-small-cell lung cancer?

**DOI:** 10.1186/s13019-024-02912-7

**Published:** 2024-06-24

**Authors:** Hidetaka Uramoto, Takaki Mizoguchi, Nozomu Motono

**Affiliations:** Department of Thoracic Surgery, Kanazawa Medical University, 1-1 Daigaku, Uchinada-machi, Kahoku-gun, Ishikawa, 920-0293 Japan

**Keywords:** NSCLC, Diabetes mellitus, Surgical resection, Postoperative complications

## Abstract

**Purpose:**

Diabetes mellitus (DM) is a common comorbidity of lung cancer. We hypothesized that severe DM is associated with increased complications after surgical resection of non-small-cell lung cancer (NSCLC).

**Methods:**

A review of our retrospective thoracic database identified 1139 consecutive surgical resections for NSCLC from 2002 to 2021. Our analysis included the exploration of clinicopathological features, perioperative variables, and surgical outcomes.

**Results:**

In addition to lung cancer, 170 patients (14.9%) had DM. The patients included 132 (77.6%) men and 38 (22.4%) women, with a median age of 72 (range, 51–93) years old. The median preoperative fasting blood glucose and HbA1c levels were 135 mg/dL (range, 57–303) and 6.9% (range, 5.1–14.8), respectively. Eighty-one patients had DM as a single comorbidity, and 89 patients had other comorbidities or a relevant medical history. A total of 144 patients were prescribed these drugs. There were 107 patients (62.9%) who consulted a specialist diabetes endocrinology department preoperatively and 118 patients (69.4%) who required sliding-scale insulin during the perioperative period. Forty-seven patients (27.6%) developed post-operative complications. No cases of bronchopleural fistula were noted. A univariate analysis showed that the sex (*p* = 0.017), body mass index (BMI) (*p* = 0.0032), surgical procedure (*p* = 0.017), surgical time (*p* = 0.002), and lymphatic invasion (*p* = 0.011) were significantly different among patients stratified by postoperative complications. A multivariate analysis showed that a low BMI (odds ratio [OR]: 0.413, 95% confidence interval [CI]: 0.196–0.870, *p* = 0.018), long surgical time (OR: 2.690, 95% CI: 1.190–6.082, *p* = 0.015), and presence of lymphatic invasion (OR: 2.849, 95% CI: 1.319–6.135, *p* = 0.007) were risk factors for postoperative complications. In contrast, severe preoperative DM did not have a significant negative effect on the incidence of postoperative complications.

**Conclusion:**

In modern respiratory surgery, severe DM does not affect the short-term outcomes under strict preoperative treatment.

## Backgroud

Lung cancer has a high mortality rate worldwide [1]. DM is a common condition worldwide [2]. Its global prevalence has risen from 4.7% in 1980 to 8.5% in 2014 among adults over 18 years old, and it is projected to be the seventh leading cause of mortality by 2030 [3]. DM is a common comorbidity among lung cancer patients [4].

A meta-analysis revealed that DM can be an independent risk factor for bronchopleural fistula (BPF) after pulmonary resection in an Asian population [5]. Another study reported that DM is associated with an increased risk of postoperative mortality [6]. In contrast, the effect of DM on the incidence of postoperative complications is not significant [7]. Therefore, the influence of DM on the postoperative course has been found to be inconsistent.

Strict preoperative glycemic control might be time-consuming, which would lead to delayed surgical treatment of NSCLC [7]. A longer waiting period for surgery is associated with an increased risk of metastasis. In other words, from a short-term perspective, it is better to postpone the surgery due to concerns about postoperative complications caused by DM. However, from a long-term perspective regarding the patient’s prognosis, it is better to perform surgical intervention without postponing it. This situation makes the decision process extremely difficult with regard to the optimal timing to perform surgery.

Strict preoperative glycemic control might be time-consuming, leading to delayed surgical treatment of NSCLC [7]. A longer waiting period for surgery is associated with an increased risk of metastasis. In other words, from a short-term perspective, it is better to postpone the surgery due to concerns about postoperative complications due to DM. However, from a long-term perspective regarding the patient’s prognosis, it is better to perform surgical intervention without postponing it. This situation makes the decision process extremely difficult regarding the optimal timing to perform surgery.

In recent years, there have been significant advances in anesthesia methods, surgical approaches, surgical materials (especially threads and needles), and perioperative management. However, despite these advances, we hypothesized that serious DM would still be of concern with regard to indications for surgery and would increase postoperative complications in severe cases.

## Patients and methods

### Patients

#### Patients

All patients included in this study provided their written informed consent for treatment after receiving a comprehensive explanation of the observational research and privacy policy. This study was conducted in accordance with the principles of the Declaration of Helsinki and approved by the ethics committee of Kanazawa Medical University (approval no. I753).

Before initiating treatment, a pretreatment evaluation was conducted, which included a review of the patient’s medical history, a thorough physical examination, a complete blood cell count, and an analysis of serum chemistry data, including electrolyte levels, enzymes reflecting the liver function, bilirubin, creatinine, and coagulation values. Chest radiography, computed tomography (CT) of the chest and upper abdomen, and bronchoscopy were also performed. A preoperative cardiovascular risk assessment was conducted based on electrocardiography (ECG) and ultrasound cardiography (UCG). Clinical staging was performed using positron emission tomography (PET) and magnetic resonance imaging (MRI) of the brain [8].

A total of 1139 NSCLC patients underwent pulmonary resection at Kanazawa Medical University between 2002 and 2021. Data collected included the sex, age, smoking history, BMI, respiratory function, comorbidity, carcinoembryonic antigen (CEA) levels, eGFR, PNI, NLR, CIPI, SUV_max_ of ^18^F-fluorodeoxyglucose PET/CT of primitive lesion, lung lobe involvement, histological tumor type, and history of adjuvant chemotherapy. The smoking history was assessed using the Brinkman Index (BI), which was calculated by multiplying the number of cigarettes smoked per day by the number of years the subject had been smoking. Comorbidity was evaluated using the Charlson Comorbidity Index [9]. Preoperative comorbidities were extracted from patient interviews and medical records. The following respiratory function parameters were measured: percent-predicted vital capacity and forced expiratory volume in 1 s as a percentage of forced vital capacity. The PNI was calculated by combining serum albumin levels with the total peripheral lymphocyte count. The NLR was defined as the neutrophil-to-lymphocyte ratio. CIPI was calculated as CEA (ng/mL) × NLR, as reported previously [10–12].

#### Perioperative management of DM

DM was included as a comorbidity if it was diagnosed according to the 1999 World Health Organization guidelines [13]. If a state in which the treatment goal of the Diabetes Standard Medical Treatment Manual 2023, published by the Japan Human Data Society of Diabetes and Related Diseases, is not achieved, the preoperative HbA1c exceeded 7%, or the fasting blood glucose value exceeded 130 mg/dL, the patient was asked to consult with the Diabetes Endocrinology Department before surgery [14]. In principle, the target blood glucose level was 200 mg/dL, and preoperative one-week hospitalization was required for patients to control preoperative blood sugar control before surgery. In principle, the target blood glucose level was 200 mg/dL, and one week of preoperative hospitalization was required to achieve preoperative blood sugar control before surgery.

#### Treatment strategy

All surgical procedures were conducted using either open thoracotomy, video-assisted thoracoscopic surgery (VATS), or a hybrid of both or robotic-assisted thoracic surgery (RATS). Systematic removal of the mediastinal lymph nodes, including contiguous fatty tissue containing lymphatic vessels, was performed for surgical procedures beyond segmental resection. In general, the bronchial stump is covered with pericardial fat tissue, for example, under certain conditions (e.g., diabetes, postoperative adjuvant platinum-based chemotherapy) or when there were concerns about bronchopleural fistula (e.g., in cases of right lower lobe resection) [15]. All surgical specimens were subjected to a pathological analysis according to the 8th edition of the TNM classification, as outlined by the International Association for the Study of Lung Cancer (IASLC).

#### Surgical factors

Operating procedures were divided into four categories: wedge resection, segmentectomy, lobectomy, and extended resection beyond lobectomy. Postoperative complications were included within 30 days of surgery. Postoperative complications were graded according to the extended Clavien–Dindo classification of surgical complications established [16] by the Japan Clinical Oncology Group [17]: grade I, conditions requiring clinical observation only; grade II, conditions requiring medical management; grade IIIa, conditions requiring medical intervention under local anesthesia; grade IIIb, conditions requiring surgical intervention; grade IVa, life-threatening complications requiring intensive-care-unit management; grade IVb, life-threatening complications involving multiple organ failure; and grade V, death.

#### Pathological factors

Data on lymphatic invasion, vascular invasion, differentiation, histological type, and pathological stage (pStage) were also collected.

#### Postoperative management and follow-up

Postoperative management and follow-up procedures involved retrospective collection of data from all patients. After the surgery, each patient was discharged from the hospital. Follow-up data were collected from outpatient departments. In principle, the patients underwent a physical examination, chest roentgenography, an analysis of blood chemistry data, and measurements of tumor marker levels every three months. Chest and abdominal CT and brain MRI were generally performed every six months until two years after surgery and then annually for five years or more [18]. Other assessments were conducted in response to the emergence of signs or symptoms indicating disease progression.

### Statistical analysis

The data were analyzed using the StatView software package (Abacus Concepts, Inc., Berkeley, CA, USA). Statistical significance was defined as *P* < 0.05. Univariate and multivariate logistic regression analyses were performed to identify factors associated with postoperative complication as an event. Odds ratios (ORs) and 95% confidence intervals (95% CIs) were calculated for each variable.

## Results

### Patient characteristics

Among them, 170 patients (14.9%) had DM in addition to lung cancer. The number of DM patients in the first (2002–2012) and second half (2013–2021) of the observation period was 33 and 137, respectively. Therefore, these patients were enrolled in this retrospective study. These cases are summarized in Table 1.

**Table 1:** Patient characteristics and summary of the preoperative factors

		*n* = 170
Sex	Male/ Female	132/ 38
Age	median (range)	72 (51–93)
BI	median (range)	920 (0-3600)
BMI	median (range)	23.9 (14.9–36.0)
%VC	median (range)	97.9 (53.4-147.5)
FEV1%	median (range)	74.1 (38.7–99.4)
CEA	median (range)	3.9 (0.7–269.0)
eGFR	median (range)	66.7 (5.9-113.9)
DM	blood glucose^a^ median (range)	135 (57–303)
	HbA1c ^b^median (range)	6.9 (5.1–14.8)
	only DM/with other comorbidities	81/89
	Type I/II	3/167
	Drug administration +/-	144/26
	Consultation with endocrine and metabolic medicine (with/without)	107/63
	Compatible with sliding (with/without)	118/52
SUV^a^	median (range)	3.9 (0.6–19.9)
Histology^b^	AD	114
	SQ	44
	Others	12
PNI	median (range)	49.9 (31.2–61.9)
NLR	median (range)	2.3 (0.5–8.5)
CIPI	median (range)	9.3 (1.0-489.6)

^a^Twenty-one cases were excluded because of no description of data. ^b^AD, adenocarcinoma SQ, squamous cell carcinoma

The patients included 132 (77.6%) men and 38 (22.4%) women, with a median age of 72 (range, 51–93) years old. The median BI, BMI, %VC, FEV1%, preoperative CEA level, and eGFR were 920, 23.9, 97.9, 74.1, 3.9, and 66.7, respectively.

Regarding the indicators related to DM, the median preoperative fasting blood glucose and preoperative HbA1c levels were 135 mg/dL (range, 57–303) and 6.9% (range, 5.1–14.8), respectively. There were 81 and 89 patients who had only DM as a complication and those who had other comorbidities or a medical history, respectively. Regarding the details of other complications and medical histories thereof, the 89 cases included 32 cases of hypertension, 2 cases of atrial fibrillation (AF), 23 cases of angina pectoris (AP), 9 cases of cerebral infarction (CI), 10 cases of chronic obstructive pulmonary disease (COPD), 6 cases of interstitial pneumonia (IP), 3 cases of bronchial asthma, 2 cases of rheumatoid arthritis (RA), 4 cases of CRF, 6 cases of colon cancer, 3 cases of prostate cancer, 2 cases of gastric cancer, 2 cases of renal cancer, and 1 case each of abdominal aortic aneurysm (AAA), malignant lymphoma, thyroid cancer, gall bladder cancer, bile duct cancer, bladder cancer, tongue cancer, arteriosclerosis obliterans (ASO), thromboangiitis, and liver cirrhosis (LC), including overlapping cases.

Most of the patients had type II DM. A total of 144 patients were prescribed the following drugs: dipeptidyl peptidase (DPP)-4 inhibitors (*n* = 80), biguanides (*n* = 54), sulfonylureas (*n* = 43), alpha-glycosidase inhibitors (*n* = 35), insulin (*n* = 30), sodium glucose transporter (SGLT)2 inhibitors (*n* = 18), thiazolidines (*n* = 13), rapid-acting insulin secretagogues (*n* = 8), and GLP-1 receptor agonists (*n* = 3), including overlapping cases.

There were 107 patients (62.9%) who consulted with a specialist in the Diabetes Endocrinology Department preoperatively and 118 (69.4%) who required sliding-scale insulin during the perioperative period. The SUV in PET for primary lung cancer was 3.9. A total of 114 patients (67.1%) were diagnosed with adenocarcinoma (AD) and 44 with squamous cell carcinoma (SQ). The median PNI, NLR, and CIPI were 49.9, 2.3, and 9.3, respectively. There was a statistically significant correlation between preoperative fasting blood glucose level and HbA1c level, whether or not the patient had visited an endocrine and metabolic medicine department, and whether or not preoperative sliding was performed.

### Operative factors

Open thoracotomy was performed in 18 patients, complete thoracoscopic surgery in 74, hybrid VATS using mini-thoracotomy with a direct view and video monitor view in 70, and RATS in 8 patients. Lobectomy was performed in 111 patients, segmentectomy in 13, bilobectomy in 3, and pneumonectomy in 2 (Table 2).

**Table 2:** Patient characteristics and summary of the intraoperative and postoperative factors

		*n* = 170
Main side of the tumor	Right	106
	Left	64
Main lobe of the tumor		
	Upper	90
	Middle	12
	Lower	68
Approach		
	Open	18
	VATS	74
	Hybrid	70
	RATS	8
Surgical procedure		
	Pneumonectomy	2
	Bilobectomy	3
	Lobectomy	111
	Segmentectomy	13
	Others	38
Surgical time		155 (27–672)
Lymphatic invasion	absent/present	119/51
Vascular invasion	absent/present	89/81
Tumor differentiation	G1/2/3/4	44/91/30/5
Pathological stage	0/I/II/III	8/128/27/7
Postoperative complication	absent/present	123/47
Grade of complication	G 1/2/3	0/16/31
Postoperative hospitalization period		10 (4-100)
Adjuvant chemotherapy	With/without	17/153

### Pathological factors

Lymphatic invasion was present in 51 patients (30.0%), and vascular invasion was present in 81 patients (47.6%). Differentiation was categorized as G1 in 44 patients, G2 in 91 patients, G3 in 30 patients, and G4 in 5 patients. The pStage was 0 in 8 patients, I in 128, II in 27, and III in 27.

### Postoperative complications

Forty-seven patients developed post-operative complications. The breakdown included 23 cases of postoperative air leak, 6 cases of AF, 4 cases of empyema, 3 cases of pneumonia, 3 cases of atelectasis, 3 cases of chylothorax, 2 cases of bronchial asthma, 2 cases of CI, 2 cases of hypoxemia with home oxygen, and 1 case each of acute respiratory distress syndrome (ARDS), empyema, urinary tract infection (UTI), ventricular tachycardia (VT), aspiration, and lower extremity artery occlusion. The grade of complications was categorized as G2 in 16 patients and G3 in 31 patients. There was some duplication of the postoperative complications.

### Univariate and multivariate analyses

First, the relationships between the clinicopathological characteristics or operative factors of the patients and postoperative complications were analyzed (Table 3). A univariate analysis showed that the sex (*p* = 0.017), BMI (*p* = 0.0032), surgical procedure (*p* = 0.017), surgical time (*p* = 0.002), and lymphatic invasion (*p* = 0.011) were significantly different among patients stratified by postoperative complications. The rate of postoperative complications was similar between severe DM cases, which require high levels of blood glucose and HbA1c and consultation with endocrine and metabolic medicine specialists/perioperative sliding, and the counterpart group. Second, a multivariate analysis showed that the BMI (odds ratio [OR], 0.41; 95% confidence interval [CI]: 0.196–0.870, *p* = 0.018), surgical time (OR: 2.690, 95% CI: 1.190–6.082, *p* = 0.015), and lymphatic invasion (OR, 2.849; 95% CI: 1.319–6.135, *p* = 0.007) were risk factors for postoperative complications.

**Table 3:** Univariate and multivariate analyses of risk factors for postoperative complication

Univariate analysis					Multivariate analysis		
Variables		OR	95% CI	*p* value	OR	95% CI	*p* value
Gender	Male	6.6	2.576–16.907	0.017	10.08	2.628–38.676	0.055
Age	< 72	1.36	0.693–2.668	0.371			
BI	< 920	1.39	0.706–2.726	0.341			
BMI	< 23.9	0.474	0.237–0.947	0.032	0.413	0.196–0.870	0.018
%VC	≥ 97.9	1.300	0.662–2.554	0.445			
FEV1%	≥ 74.1	2.111	1.056–4.221	0.032			
CEA	< 3.9	0.924	0.471–1.812	0.818			
eGFR	≥ 66.7	1.155	0.589–2.265	0.675			
Glu	< 135	1.006	0.514–1.971	0.986			
HbA1c	< 6.9	1.316	0.671–2.581	0.425			
Comorbidities	Present	1.169	0.597–2.291	0.648			
Drug	administration	1.150	0.484–2.733	0.752			
Consultation	with	0.835	0.413–1.732	0.606			
sliding	Compatible	0.824	0.392–1.732	0.606			
PNI	< 49.9	0.894	0.456–1.754	0.745			
NLR	≥ 2.3	0.838	0.427–1.643	0.607			
CIPI	< 9.3	1.388	0.706–2.726	0.341			
Main side of the tumor	Right	1.176	0.6591–2.342	0.645			
Main lobe of the tumor	Lower	0.682	0.338–1.374	0.280			
Approach	Open/ Hybrid	0.572	0.288–1.137	0.108			
Surgical procedure	equal to or greater than lobectomy	3.942	1.552–10.012	0.001	0.750	0.338–1.665	0.479
Surgical time	< 155	2.913	1.432–5.925	0.002	2.690	1.190–6.082	0.015
Histology	AD	0.634	0.315–1.275	0.204			
Lymphatic invasion	Present	2.506	1.235–5.076	0.011	2.849	1.319–6.135	0.007
Vascular invasion	Present	0.736	0.375–1.443	0.371			
Tumor differentiation	G 3/4	1.491	0672-3.308	0.332			
Pathological stage	II/ III	1.574	0.706–3.503	0.274			
Adjuvant chemotherapy	administration	0.908	0.302–2.734	0865			

AD, adenocarcinoma

## Discussion

Comorbidity of preoperative diabetes has long been discussed in relation to the development of postoperative complications. DM is generally associated with microcirculation disorders, which are considered to cause postsurgical complications [19]. In contrast, Komatsu et al. reported that preoperative DM does not influence acute-phase postoperative recovery [7]. Wang et al. also reported that the no-DM group (*n* = 643) and the DM group (*n* = 126) were similar in terms of postoperative complications, with no significant differences noted [20].

We previously verified the validity of the risk calculator in the National Clinical Database (NCD) of 585 patients who underwent pulmonary resection for NSCLC. It was reported that the coexistence of DM did not affect the occurrence of postoperative complications (*p* = 0.49) [21]. Microvascular damage renders patients susceptible to infection, delays wound healing in patients with DM, and often causes serious postoperative complications. Thus, the appropriateness of drawing a line between more severe and less severe DM remains unclear.

Strict blood sugar control for severe DM cases requires long-term management, and as a result, surgery is postponed, resulting in lymph node metastasis, which can put the cart before the horse in terms of the patient prognosis. However, if a poorly controlled patient is operated on too quickly and suture failure occurs, it can be fatal. Therefore, patients with DM who have poor glycemic control may develop severe postoperative complications. However, in contrast to our hypothesis, severe DM does not increase the incidence of postoperative complications after surgical resection of NSCLC. It is noteworthy that there was no occurrence of BPF, suture failure, or surgical site infection (SSI) in our series. Therefore, serious management may not be as important as we think in today’s world, where dramatic advances in medical care have been achieved.

In patients with severe DM, it often takes several weeks to successfully control the blood sugar levels before surgery, leading to delayed surgical treatment for NSCLC, and does not increase postoperative complications, which would be important information for surgeons. However, 47 patients in the present study developed postoperative complications, including infectious diseases, such as pneumonia and empyema. Therefore, careful perioperative management of this condition is essential.

It is well known that patients with a low BMI and a high pack-year count have a higher rate of pulmonary complications than others [22]. A low BMI was a risk factor for postoperative complications in this study. However, there was no statistically significant association between a low BMI and high BI scores. A thin body build in patients with DM may reflect a reduced whole-body muscle mass and suggest that such patients are less able to withstand surgery, regardless of their smoking status.

A long surgical time is a risk factor for postoperative complications. This is easy to accept because surgical time is directly linked to surgical invasiveness, and it can be imagined that complications will result. One unusual discovery in the present study was that lymphatic infiltration is a risk factor for postoperative complications. Recently, Yuan et al. reported that the identified core genes of NSCLC and DM are associated with dysregulated immune cells, which provides a potential research avenue for diagnosing and treating lung cancer combined with DM [23]. In other words, patients with DM have shared genetic features and immune infiltration processes that are different from those of patients without DM. It can be inferred that the oncological features of certain conditions lead to an immunocompromised state. Originally, glycolysis was considered important for tumor metabolism and growth, manifesting as a heightened glucose uptake rate [4]. We previously reported that smoking history, vascular invasion, and pStage, but not postoperative complications, were identified as significant prognostic factors in a multivariate analysis of 463 patients with NSCLC [24]. Recently, however, we showed the clinical relevance of SGLT2 in resected lung adenocarcinoma, and the expression of SGLT2 was more frequently detected in advanced-stage and more aggressive adenocarcinomas with aggressive biological behavior than in their counterparts. A survival analysis revealed that a strong SGLT2 expression was associated with a poorer recurrence-free survival. The SGLT2 expression predicts postoperative recurrence in patients with lung adenocarcinoma [25]. Therefore, postoperative outcomes may vary depending on the efficacy of the drug and the biological characteristics of the tumor.

The present study was associated with several limitations, including its retrospective design, and long observational term under the single-institutional setting. Furthermore, the study lacked data from non-DM patients and postoperative daily glucose values. Notwithstanding these limitations, severe DM did not increase the incidence of postoperative complications after surgical resection of NSCLC under strict preoperative treatment.

## Conclusions

In the present study, a low BMI, long surgical time, and lymphatic invasion were risk factors for postoperative complications. However, severe DM does not increase the incidence of postoperative complications after surgical resection of NSCLC. In modern respiratory surgery, severe DM does not affect short-term outcomes.

## Data Availability

No datasets were generated or analysed during the current study.
